# Comparison of the effectiveness of autofluorescence devices in the detection of parathyroid glands during thyroid operations: a propensity score matched analysis (PSM)

**DOI:** 10.3389/fendo.2026.1846397

**Published:** 2026-07-06

**Authors:** Nikolaos Voloudakis, Eleni Lazaridou, Stefanos Atmatzidis, Evangelia Bellou, Maria Velikoudi, Anestis Basios, Basileios Papaziogas, Ioannis Koutelidakis

**Affiliations:** Second Surgical Department, G. Gennimatas General Hospital of Thessaloniki, Aristotle University of Thessaloniki, Thessaloniki, Greece

**Keywords:** autofluorescence, intraoperative preservation, naked eye, NIRAF, parathyroid, thyroid surgery

## Abstract

**Introduction:**

In recent years, an abundance of evidence has supported the use of specialized autofluorescence devices to assist in detecting parathyroid glands (PGs) during thyroid surgery. This study compares the “naked eye” (NE) method with an alternative device (IMAGE1 S™ Rubina^®^ -R), primarily designed for ICG (Indocyanine Green) angiography, and a standard autofluorescence device (Fluobeam^®^ Fluoptics LX - F) in terms of parathyroid identification.

**Methods:**

From 01/01/2024 to 31/08/2025, data from all patients undergoing thyroid-related surgery were collected from a prospectively maintained database. Adult patients who had total thyroidectomy with or without bilateral central neck dissection were included. Patients with concurrent hyperparathyroidism or renal insufficiency were excluded. In the intervention groups, the IMAGE1 S™ Rubina^®^ camera and Fluobeam^®^ LX were used, while no adjunct devices were employed in the control group. Patients were retrospectively matched based on indication for surgery, extent of operation, and operating surgeon. The primary endpoint was the number of parathyroid glands identified intraoperatively.

**Results:**

292 patients were included in the study. Propensity Score Matching yielded 37 patients per group, with no differences in demographic or clinical features. Ordinal logistic regression for the number of parathyroid glands identified showed higher odds of better gland identification with Rubina^®^ versus Naked Eye (OR 2.56, 95% CI 1.10–5.97) and with Fluobeam^®^ versus Naked Eye (OR 4.12, 95% CI 1.71–9.95), while Fluobeam^®^ did not differ significantly from Rubina^®^ (OR 1.67, 95% CI 0.72–3.88). Postoperative PTH levels were 26.63 ± 7.02 pg/ml in the NE group, 30.02 ± 5.70 pg/ml in the Rubina^®^ group, and 33.09 ± 5.32 pg/ml in the Fluobeam^®^ group (NE vs R: p = 0.046, NE vs F: p = 0.0001, R vs F: p = 0.079). No differences were found in postoperative calcium levels, hypoparathyroidism rates, the number of parathyroid glands in the pathology report, or the need for intraoperative PG autotransplantation.

**Conclusion:**

Detection rates of parathyroid glands improved with the use of both autofluorescence devices compared to the naked eye, while there was no significant decrease in postoperative hypoparathyroidism rates.

## Introduction

Thyroidectomy is the most common and among the safest endocrine operations. Over the years, the procedure has been technically refined, leading to excellent postoperative and oncological outcomes when performed at experienced centers. However, complications can occur and may impact the patient’s quality of life or, less frequently, be life-threatening. Edafe et al., in their systematic review, identified postsurgical hypocalcemia as a major consequence of thyroid surgery, with an incidence of 19–38 percent for transient cases and 0–3 percent for permanent cases (1). Recognizing and preserving the parathyroid glands (PGs) during surgery is crucial but can be difficult even for high-volume thyroid surgeons. Challenges mainly arise from the small size of PGs, their camouflaged appearance amid fatty and thyroid tissue, or their location within the thyroid gland itself (2). Postoperative hypocalcemia may result from surgical injury, accidental resection, or unintentional damage to healthy parathyroid glands’ blood supply. Inadvertent damage or removal of PGs during surgery can cause lifelong issues with calcium regulation and potentially lead to renal sequelae or increased morbidity (2–5). Consequently, substantial research has focused on developing auxiliary devices to better identify and protect PGs, with the aim of reducing the incidence of postoperative hypoparathyroidism.

Among the techniques investigated, autofluorescence (AF) has yielded the most promising results and is the focus of recent literature. Fluorescence is an intrinsic property of certain molecules that occurs when light is absorbed, inducing electron transitions that lead to photon emission at a lower energy and longer wavelength. Such a property exists in PGs as well, due to endogenous fluorophores that emit a fluorescent signal upon exposure to near-infrared (NIR) light (6). Although several possible fluorophores have been suggested for PGs autofluorescence, including the calcium-sensing receptor, none has been proven to date (7).

In 2011, Paras et al. were the first to report the autofluorescent properties of PGs in the NIR spectrum, demonstrating that these glands emit light with a peak at approximately 820 nm upon excitation at 785 nm. Their findings indicated that parathyroid tissue exhibits autofluorescence up to 11 times that of the thyroid gland. Based on these results, the authors concluded that PGs can be reliably, nonintrusively differentiated from surrounding structures of the neck (8), regardless of disease or state of the PG (7).

Since then, the ability of AF devices to detect PGs has been repeatedly demonstrated and is nowadays an established concept (9–12). Devices that use NIR-AF technology to localize PGs in real time are either probe- or image-based. The only device in the first category with FDA approval is the Medtronic^©^ PTeye device (AiBiomed, Santa Barbara, CA) (13). Among image-based tools, Fluobeam-800 and Fluobeam-LX (Fluoptics^©^, Grenoble, France) are FDA approved. The latter consists of a camera system that emits NIR light and a screen that displays the surgical field of interest (14).

Apart from AF described above, another modality used in thyroid surgery is immunofluorescence, primarily with the use of fluorescence dyes, such as indocyanine green (ICG). ICG fluorescence has been used in various surgical fields to evaluate tissue perfusion and guide procedures, particularly in HPB surgery, during bowel anastomosis or lymphatic interventions (6). ICG, when injected intravenously, binds to plasma proteins and remains within the vascular space, acting as a contrast agent (14). ICG can visualize the vascular network of PGs and, therefore, can be used as an adjunct in their intraoperative detection and preservation (15). Specifically, in plasma, ICG exhibits an absorption peak at 807 nm and an emission peak at 822 nm, both within the NIR window (16). This spectrum is quite close to that of PGs’ autofluorescence, making tools primarily designed for ICG angiography potentially useful for PGs visualization as well.

In the literature, few publications use devices designed for ICG angiography to identify PGs via autofluorescence alone. Among them are Karl Storz Opal-1 (Karl Storz^©^, Germany) (17, 18) and PDE Neo II (Hamamatsu^©^, Japan) (14). However, their efficiency has never been compared with that of dedicated autofluorescence devices.

This study aims to compare label-free optical devices that use intrinsic PGs properties with naked-eye PG visualization alone during thyroid operations. Specifically, it examined whether there is a statistically significant difference in the rate of PG identification between an ICG angiography device and a dedicated PG detection device.

## Methods

This retrospective cohort study included patients who underwent thyroid-related surgery between January 2024 and August 2025. Data were extracted from a prospectively maintained departmental database. This was a single-center study, and all procedures were performed by two high-volume endocrine surgeons using PG detection probes (19). The same surgical technique and energy devices were used in every case, along with intraoperative neuromonitoring (IONM).

**Figure 1:**
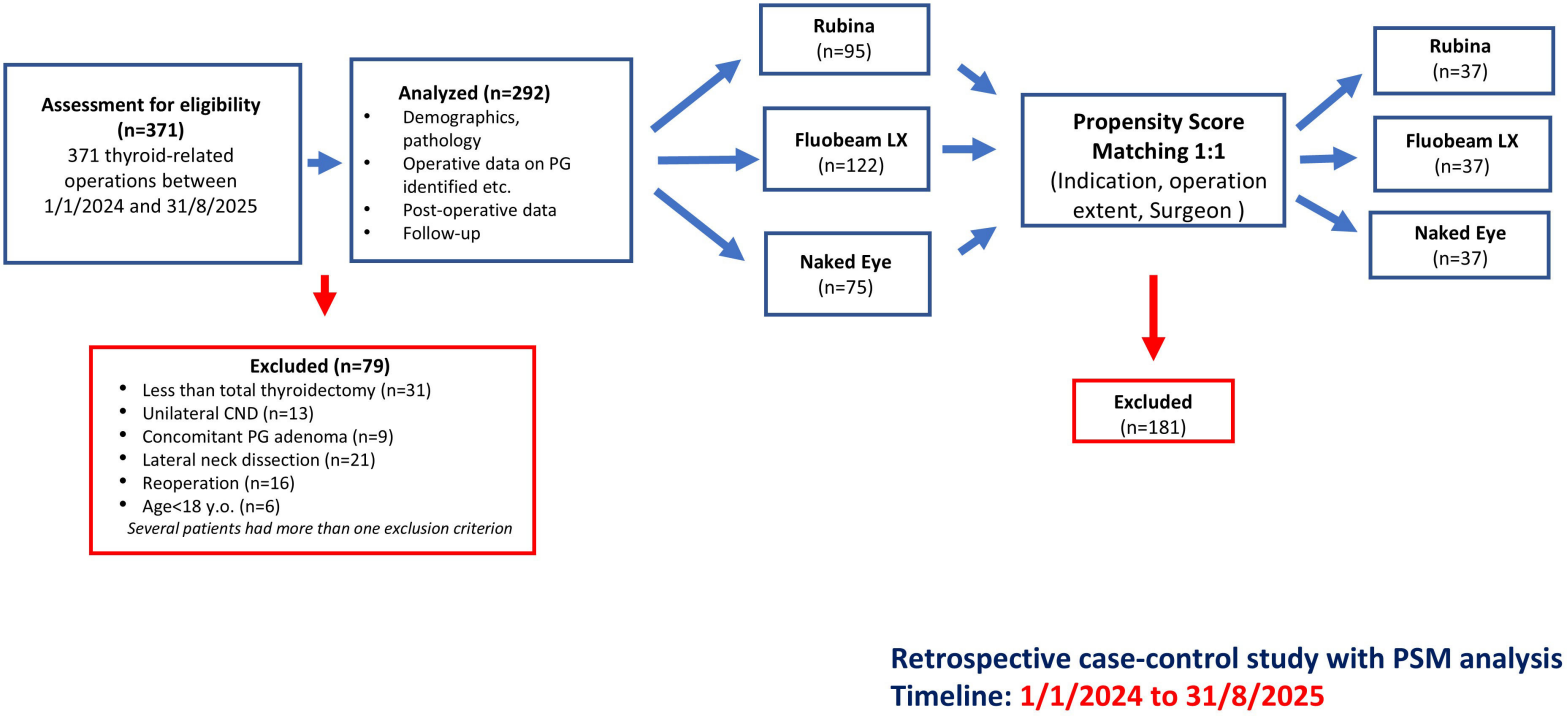
Study flowchart.

The study included all adult patients who underwent total thyroidectomy alone or total thyroidectomy and bilateral central neck dissection (CND). Patients with a history of cervical endocrine surgery or those who underwent lateral neck dissection were excluded. Thirteen patients that underwent only unilateral CND for frozen section examination purposes were excluded. Non-adult patients and individuals with concomitant parathyroid disease or renal insufficiency were also excluded. The study flowchart is shown in **Figure 1**.

In the control group, parathyroid glands were detected visually with the naked eye alone. Specifically, traditional identification relied on the surgeon’s experience, color differences, and recognition of cervical anatomical landmarks. After visually identifying the parathyroid glands, careful dissection was performed to preserve their blood supply. If the glands were not visible in the immediate surgical field, no further searching was done. Following thyroid removal, the specimen was reexamined for possible parathyroid tissue. Any glands found were reimplanted into the sternocleidomastoid muscle, following frozen-section examination in cases of suspected or confirmed malignancy.

**Figure 2:**
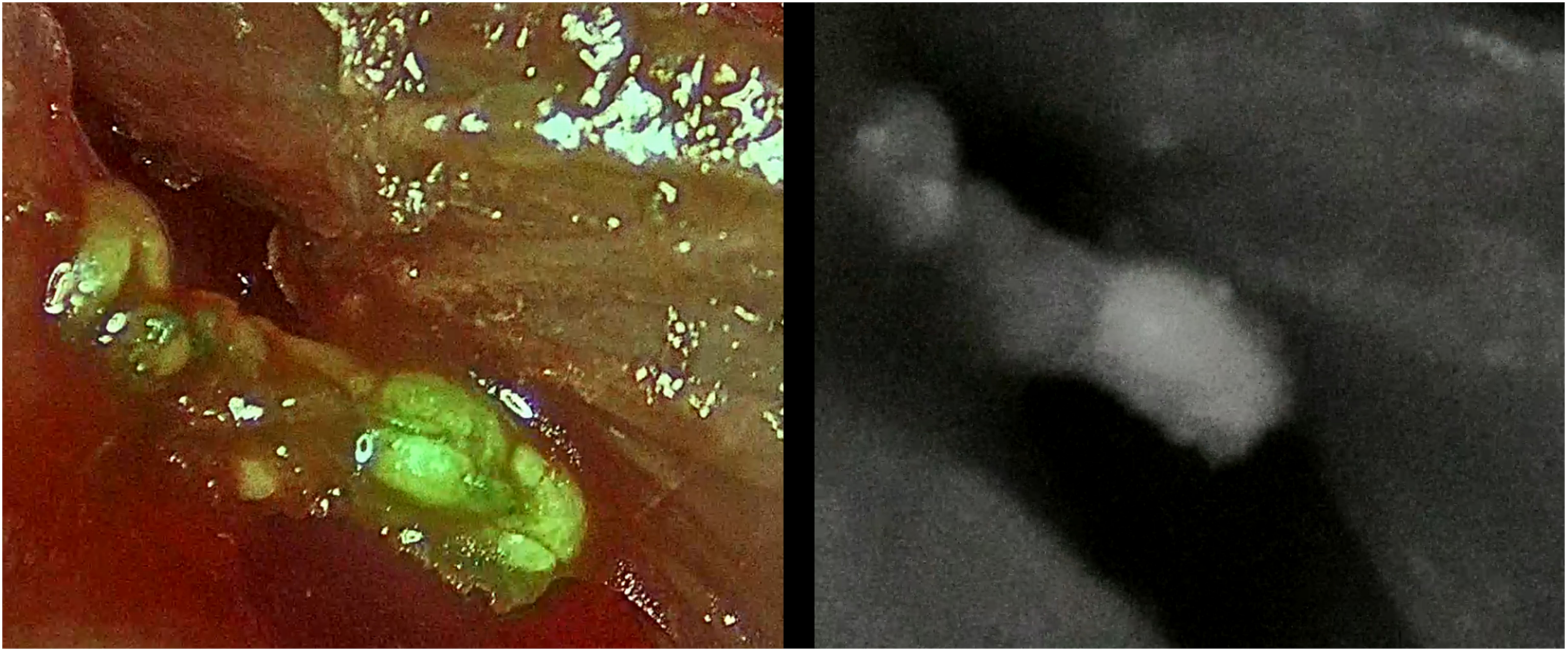
An upper parathyroid gland as seen in overlay **(A)** and Monochromatic mode **(B)**.

In the second group, only the Storz Near-Infrared Range/Indocyanine Green (NIR/ICG) imaging system (Karl Storz, Tuttlingen, Germany) was used for PG detection. This camera system is typically employed for ICG angiography during open or endoscopic procedures. Its 4K high-definition IMAGE1 S™ RUBINA™ camera head (3849 × 2160 pixels) features two image sensors: one for white-light detection and another for NIR/ICG signals. The modes used were overlay, which combines a white light image with NIR/ICG, and monochromatic. The POWER LED RUBINA™ light source features dual light-emitting diodes, one for white light and one for a broad NIR wavelength (around 800nm) (**Figure 2**). After mobilization of each lobe, the surgeon detected and exposed any possible parathyroid tissue; the PGs, along with surrounding structures, were illuminated with NIR light after the theater lights were turned off. The camera tip was held approximately 5cm from the operative field. Once fluorescent PGs were recognized—appearing greenish in overlay mode and white in monochromatic mode—the operation resumed in the standard fashion.

**Figure 3:**
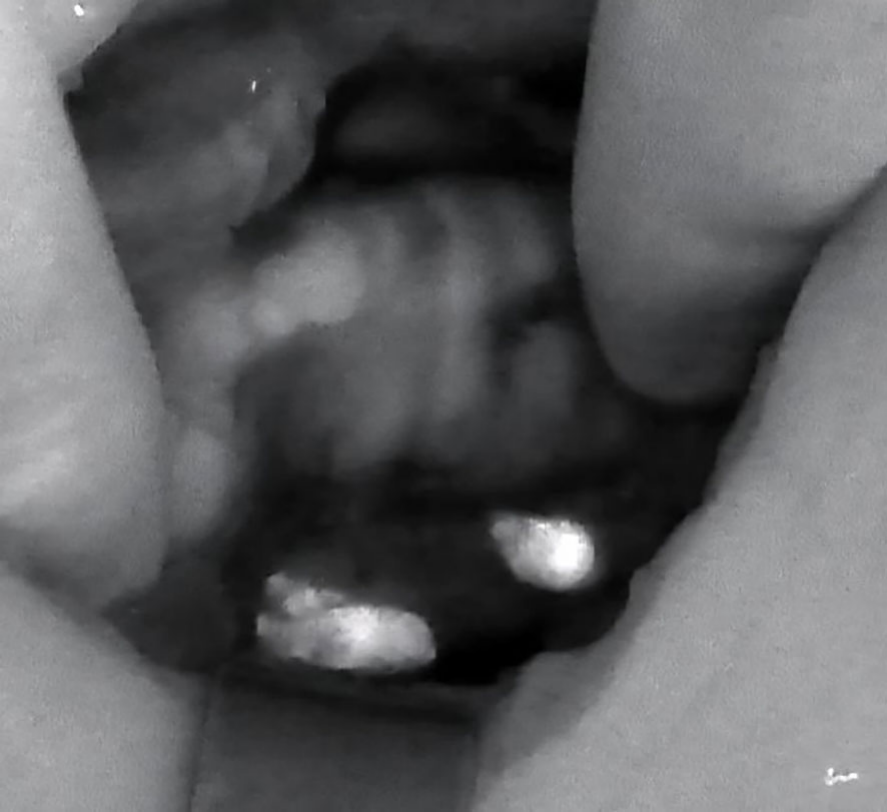
Post-thyroidectomy image of the right thyroid bed depicting both parathyroid glands.

In the third group, only Fluobeam^®^ LX (Fluoptics, Grenoble, France) was used to visualize PGs intraoperatively (**Figure 3**). The process was identical to that of the second group, except that the camera was held approximately 10cm from the field, as per the manufacturer’s instructions (**Figure 3**).

In all cases where adjuncts were used, the surgeon did not rely exclusively on them to decide whether any tissue was PG, but rather used them as visual feedback to support his/her anticipation or to further explore fluorescent tissues he/she might have overlooked.

**Figure 4:**
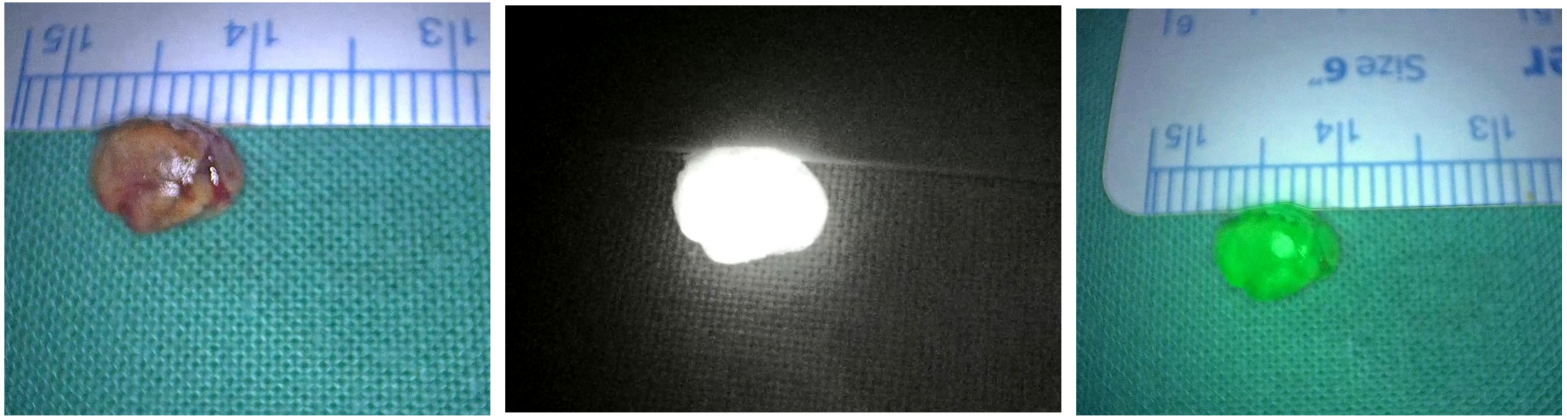
A parathyroid gland accidentally removed during central neck dissection as seen in white light **(A)**, Overlay mode **(B)**, and Monochromatic **(C)**.

Specimens from both intervention groups were evaluated using each device, and if accidental PG removal occurred, reimplantation was carried out as in the control group (**Figure 4**). No ICG angiography was performed in any case. This study explored the adequacy of the Rubina™ to detect the PGs with autofluorescent properties alone.

Device selection, or lack thereof, in the department’s practice during that timeframe was based solely on availability. To mitigate potential selection bias in this retrospective study, propensity score matching (PSM) was performed. The propensity score was estimated using indication for surgery (operated for malignancy/suspicious for malignancy or not), operation extent (TT or TT and CND), and surgeon as covariates, and patients were matched using an optimal one-to-one matching algorithm. A caliper width of 1 was applied. PSM was performed using XLSTAT statistical and data analysis software (Addinsoft, New York, NY, USA) (20).

The distribution of variables was evaluated using the Shapiro-Wilk test. Continuous variables are presented as mean (± standard deviation) or median (interquartile range), depending on their distribution, unless otherwise specified. Differences between groups were compared using one-way ANOVA for normally distributed data and the Kruskal-Wallis test for non-normally distributed data. When statistically significant differences were identified, *post-hoc* pairwise comparisons were performed using Tukey’s test for ANOVA and Dunn’s test with Bonferroni correction for Kruskal–Wallis analyses. For the primary endpoint of parathyroid glands identified, an ordinal logistic regression (proportional-odds) model was used, and expressed as odds ratios (ORs) with 95% confidence intervals (CIs). Categorical variables were analyzed using chi-square or Fisher’s exact test, as appropriate. Data analysis was conducted with IBM SPSS Statistics for Windows, Version 25.0 (Armonk, NY: IBM Corp). All tests were two-tailed, with a significance level of p<0.05.

The primary outcome was the number of *in vivo* PGs identified. Secondary outcomes included the number of PGs auto-transplanted intraoperatively and the number of PGs reported in the pathology report. Clinical and laboratory outcome measures included calcium and parathyroid hormone (PTH) levels on the first postoperative day, as well as the frequency of hypoparathyroidism (transient or permanent). For this study, transient hypoparathyroidism was defined as a PTH level below the normal laboratory range (<15 pg/mL) on the first post-op day, and permanent hypoparathyroidism was defined as the need for supplementation (regardless of PTH value or lack of discontinuation attempt) beyond 6 months after surgery.

The study was conducted in accordance with local legislation and institutional requirements. Given the retrospective, observational, and non-interventional design of the study, the Scientific Board of the General Hospital G. Gennimatas of Thessaloniki waived the requirement for further informed consent (Article No. 9/14-01-2026).

## Results

Out of 371 operations, 292 cases were included. Seventy-five were performed without any adjuncts for PGs recognition; 95 cases used the Rubina^®^ camera, and the remaining 122 cases used the Fluobeam^®^ LX. Propensity score matching was used to select 37 patients from each group for further analysis. Patients’ demographic and clinical data are summarized in **Table 1**. There were no significant differences in age, sex distribution, body mass index, extent of surgery, indication for surgery, or baseline calcium and PTH levels following matching. Concerning the final pathology thyroid cancer cases, all patients had papillary thyroid cancer in the matched groups, except for two medullary cases (one in the Fluobeam^®^ LX and one in the naked-eye group).

**Table 1:** Included patients’ characteristics and comparative analysis between groups.

	Naked eye (n=37)	Rubina (n=37)	Fluobeam (n=37)	p
Age (median, IQR) years	51 (42-64)	48 (44-61)	52 (43 -62)	0.481
Sex (male)	10 (27%)	9 (24%)	8 (22%)	0.863
BMI	28.9 ± 3.4	30.2 ± 2.9	29.5 ± 3.3	0.223
Extent of Operation				
TT^a^	28 (76%)	28 (76%)	28 (76%)	1
TT + CND^b^	9 (24%)	9 (24%)	9 (24%)	
Malignancy^c^	12 (32%)	12 (32%)	12 (32%)	1
Graves’ Disease	1 (3%)	1 (3%)	2 (5%)	1
Other Thyroiditis	5 (14%)	4 (11%)	4 (11%)	1
Calcium baseline, mg/dl (mean, SD)	9.14 ± 0.47	9.01 ± 0.61	9.12 ± 0.57	0.555
PTH baseline, pg/ml (mean, SD)	44.50 ± 14.1	47.3 ± 15.02	49.24 ± 17.01	0.416

^a^ TT, Total thyroidectomy

^b^ CND, Central neck dissection

^c^ Indication for surgery: malignancy or suspicious for malignancy.

As shown in **Table 2**, ordinal logistic regression indicated that, compared with Naked Eye, Rubina^®^ was associated with statistically significant higher odds of a better parathyroid-gland identification category (OR 2.56, 95% CI 1.10–5.97), and Fluobeam^®^ was associated with even greater odds (OR 4.12, 95% CI 1.71–9.95). The difference between Fluobeam^®^ and Rubina^®^ was not statistically significant (OR 1.67, 95% CI 0.72–3.88).

**Table 2:** Parathyroid glands identification and preservation outcomes.

	Naked Eye (n=37)	Rubina (n=37)	Fluobeam (n=37)	p	*Post-hoc*
Parathyroid Glands identified^*^	2.38 ± 1.11	2.92 ± 1.01	3.16 ± 0.99	0.0046	NE vs R: OR 2.56 (95% CI 1.10-5.97, p = 0.03) NE vs F: OR 4.12 (95% CI 1.71-9.95, p =0.002) R vs F: OR 1.67 (95% CI 0.72-3.88, p = 0.235)
PGs Identified per patient	Total no: 88	Total no: 108	Total no: 117		
0	2 (5%)	1 (3%)	1 (3%)		
1	6 (16%)	2 (5%)	1 (3%)		
2	11 (30%)	8 (22%)	6 (16%)		
3	12 (32%)	14 (38%)	12 (32%)		
4	6 (16%)	12 (32%)	17 (46%)		
Patients with Autotransplantation	4 (11%)	5 (14%)	7 (19%)	0.706	
Patients with PGs in final Histology	6 (16%)	3 (8%)	5 (14%)	0.676	

*The distribution is non-parametric, and statistical tests were done accordingly. Data is shown as means and SD for better visual comprehension.

Postoperative biochemical outcomes are summarized in **Table 3**. First postoperative PTH levels differed significantly among groups (p = 0.0001), with the Fluobeam^®^ group demonstrating statistically higher PTH levels compared with the naked-eye group (33.09 ± 5.32 vs 26.63 ± 7.02 pg/ml, p = 0.0001). A significant difference was also observed between the Rubina^®^ group and the naked-eye group (30.02 ± 5.70 vs 26.63 ± 7.02, p=0.046). No significant differences were observed between the Rubina^®^ and the Fluobeam group^®^. First postoperative calcium levels were similar among groups (p = 0.273).

**Table 3:** Clinical and laboratory patient outcomes.

	Naked Eye (n=37)	Rubina (n=37)	Fluobeam (n=37)	p	*Post-hoc*
First post-op Calcium mg/dl (mean, SD)	8.58 ± 0.43	8.43 ± 0.54	8.62 ± 0.61	0.273	
First post-op PTH pg/ml (mean, SD)	26.63 ± 7.02	30.02 ± 5.70	33.09 ± 5.32	0.0001	NE vs R: p = 0.046 NE vs F: p = 0.0001 R vs F: p = 0.079
Transient Hypoparathyroidism	10 (27%)	7 (19%)	6 (16%)	0.490	
Symptomatic Hypoparathyroidism	1 (3%)	1 (3%)	0 (3%)	1	
Permanent Hypoparathyroidism	1 (3%)	0 (0%)	1 (3%)	1	

Regarding clinical outcomes, transient hypoparathyroidism occurred in 27% of the control group, while NIRAF groups had 19% (Rubina^®^) and 16% (Fluobeam^®^), respectively, without yielding a statistical difference All patients typically recovered within a few weeks, with 3% in the Fluobeam and Naked-eye group and none in the Rubina group requiring supplementation at 6 months. Notably, the rate of symptomatic hypoparathyroidism was identical in the naked-eye group and the Rubina^®^ camera group, while none of the Fluobeam^®^ group exhibited symptoms.

## Discussion

In this study, the performance of a device not specifically designed for PG autofluorescence was compared with that of a dedicated commercial device and visual identification alone. The non-dedicated device was statistically non-inferior to the dedicated device on most measured parameters, and both yielded significantly better outcomes than visual identification alone in parathyroid gland identification.

It is well established that autofluorescence alone, or when combined with ICG angiography, improves the outcomes of PGs identification and preservation. Two recent large randomized trials support the use of image-based NIRAF technology during thyroid surgery. Specifically, a single-center trial by Dip et al. demonstrated that the use of NIRAF reduced hypocalcemia rates by 90% compared to the control group, in which thyroidectomy was performed under white light (21). Additionally, the PARAFLUO multicentric RCT, involving 245 patients, exhibited a higher rate of parathyroid gland identification and preservation, along with a lower incidence of postoperative hypocalcemia in the NIRAF group (22). Yin et al. used NIRAF in a randomized controlled trial to identify parathyroid glands and, combined with ICG, to evaluate their vascularity *in situ*. They reported a significant decrease in transient hypoparathyroidism and more precise parathyroid gland perfusion compared to NE (23).

Due to NIRAF-based identification of PGs being a noninvasive, label-free method, its popularity as an alternative to the NE approach alone during thyroid surgery has surged (14).

The current study’s results align with the previously mentioned literature. Image-based NIRAF assistance significantly outperformed the standard naked-eye technique in several outcomes, or showed a favorable trend. There was a significant difference in intraoperative PGs identified and in PTH levels postoperatively when adjunct devices were used. Our experience with IMAGE1 S™ Rubina^®^ NIR/ICG endoscopic imaging as a supplementary tool, rather than a dedicated device, demonstrated its effectiveness in assisting with PGs recognition and preservation.

Certain optical cameras that incorporate NIRAF technology, originally designed for non-endocrine procedures, can be used in thyroid surgery because the spectral features of ICG and PG autofluorescence are closely related. Since these devices are available at most centers, they could be an affordable alternative, helping novice surgeons or those with low case volumes improve their outcomes. However, in the present study, the non-dedicated device did not outperform the dedicated device on any measured parameter. In fact, the Fluobeam group had a positive, non-significant, trend concerning PG identification and post-op PTH levels when compared to the Rubina group (**Table 3**). Consequently, the purchase of a dedicated machine can be postponed until deemed cost-efficient, but high-volume centers would probably benefit more from a dedicated device.

Furthermore, there are distinct disadvantages to commercial NIR imaging systems designed for ICG fluorescence compared to those designed for PG AF. They are not as user-friendly as systems dedicated to endocrine surgery. Laparoscopic cameras can be harder to handle than dedicated handheld devices due to their longer length. There is also a learning curve involved in becoming comfortable with the appropriate distance between the camera and the target tissue. Since there must be a smaller distance between the camera and the target tissue, signals from slightly ectopic PGs or those located deeper in the field might not always be detected due to NIRAF penetration limits. This can sometimes lead to false negative results. In addition, the “PG mapping” after mobilizing each lobe is inferior in the Rubina^®^ compared to the Fluobeam^®^ LX device due to the need for a shorter distance between the camera and the target tissue, as well as focus correction. The above are probably the reasons why the Rubina^®^ group was less effective, although not statistically so, for field mapping and PG identification in comparison to Fluobeam^®^.

Overall, this study’s basic weakness lies in its retrospective design. An attempt was made to overcome this limitation to some extent by using propensity score matching to achieve pseudorandomization and limit any potential selection bias. Although the matching process improved balance across clinically relevant procedural and surgeon-related covariates, this reduction in sample size markedly limited the power of the study for secondary endpoints such as hypoparathyroidism (*post-hoc* power: approximately 21%, α=00.5). In addition, this study does not account for the potential learning curve associated with the application of autofluorescence technology in thyroid surgery. The surgical team had at least 2 years of experience with each device before the study timeframe, and the outcomes might not be repeatable for surgeons applying them for the first time. Lastly, the outcomes of unilateral CND were not appraised in this study.

## Conclusion

In this study, both a dedicated and a non-dedicated NIRAF device facilitated PG recognition significantly over naked-eye alone. Unfortunately, the study was not adequately powered to yield any significant difference in clinical outcomes such as transient hypoparathyroidism. There was a positive trend of the dedicated device over the non-dedicated device in most parameters measured, although the difference was not significant in this sample. Thus, such adjuncts could be used as an alternative in centers without access to a dedicated device.

## Data Availability

The raw data supporting the conclusions of this article will be made available by the authors, without undue reservation.
